# Infectivity of an emerging fish parasite *Gyrodactylus sprostonae* in juvenile carp (*Cyprinus carpio*)

**DOI:** 10.1002/aff2.170

**Published:** 2024-05-28

**Authors:** Numair Masud, Chris Williams, Joanna James, Jo Cable

**Affiliations:** 1 School of Biosciences, Cardiff University, Cardiff, UK; 2 Environment Agency, Huntingdon, UK; 3 APEM Ltd, Cambridge, UK

**Keywords:** carp, emerging infectious diseases, gyrodactylids

## Abstract

The gill monogenean ectoparasite *Gyrodactylus sprostonae* is an emerging pathogen within recreational UK carp fisheries, and a major cause of mortality in adult carp. This gill infection has only been noted in adult carp and not in juveniles, and no reports of its fundamental infection dynamics exist. The current study compared the infective potential of *G. sprostonae* between adult and juvenile common carp (*Cyprinus carpio*) and quantified parasite numbers on the body surface and gills of infected juveniles at two temperatures (14 and 24°C). *G. sprostonae* was able to infect the body surface and the gills of juvenile carp, and temperature significantly impacted the duration of infection and number of parasites. Interestingly, however, all juveniles under both temperature treatments lost their infections after a maximum of 40 days, with no observed clinical signs of parasitaemia or mortalities. This study therefore indicates that *G. sprostonae* does not appear to be harmful to juvenile common carp, and we discuss why this infection only seems to impact prised adult carp in the UK.

## 1 Background and Aim

In 1962, *Gyrodactylus sprostonae* was identified on the gills of common carp (*Cyprinus carpio*) in China (Mo-en, 1962). Since then, this species has been reported on common carp in other Asian countries, including, Syria and Iran and European countries, specifically Hungary, Germany (Barzegar et al., 2018), the UK (EA pers. comm.) and in South Africa (see Maduenyane et al., 2023). In the UK, this parasite has been the main cause of significant mortalities of large carp specimens within recreational fisheries (Environment Agency UK Gov. Report, 2020). Clinical signs include gill hyperplasia, respiratory distress and mortality from hypoxia (described by Mattheis & Glaser, 1970), but the infection dynamics of this parasite remain poorly reported. Notably, *G. sprostonae* infections or associated mortalities have not been reported in juvenile carp within managed stocks. Here, we tested the infectivity of *G. sprostonae* to juvenile common carp (*C. carpio*), including studying the transmission potential between adult and juvenile fish and the impact of temperature on infection dynamics.

## 2 Materials and Methods

### 2.1 Carp and parasite source and maintenance

Juvenile carp (<1 year old, SL 700–900 mm, *C. carpio, n* = 250) from V.S. Fisheries Ltd. and kept at UK outdoor ambient temperatures were acclimatised to laboratory conditions at two temperatures (14 and 24 ± 0.5°C) for a week prior to infections. As no information currently exists on the impact of temperature on this parasitic infection, these two temperatures were chosen to maximise the possibility of detecting any effects, while also being within the temperature range for common carp (Kottelat & Freyhof, 2007). All juvenile carp were screened using the technique of King and Cable (2007) to confirm they were monogenean free prior to experimental use. In each temperature treatment, carp were randomly distributed into two groups, those destined for experimental infections (*n* = 89) or uninfected control fish (*n* = 24) at a density of 1 fish per 2.5 L under 12 h dark:12 h light cycles and fed twice daily on commercial trout pellets. A 50% water change occurred weekly for the juvenile fish. Adult donor carp (65–75 cm standard length), displaying clinical signs of gill infections (lethargy, increased opercular beat rate, inflamed gills and surface breathing) were obtained from a UK recreational fishery with a history of *G. sprostonae* infections. Adult fish were maintained individually in 400 L tanks constantly aerated with a 50% daily water change. Commercial water quality testing kits were used to ensure that pH, ammonia, nitrites and nitrates were within healthy ranges (i.e. pH: 6–7, ammonia: undetectable, nitrites: <0.1 mg/L, nitrates: <30 mg/L).

### 2.2 Experimental infections

For all experimental infection protocols, we utilised gills obtained from adult carp that had been humanely euthanised via cranial destruction (Home Office Schedule 1 approved procedure). Parasite identification was confirmed with mucus scrapings of adult donor carps prior to experimental infection by examination of opisthaptor morphology using an Olympus compound microscope at x100 magnification (see Paladini et al., 2009 for details). Subsequently, three protocols were utilised to study the infectivity of *G. sprostonae* to juvenile carp. Note, for all protocols below, gills were obtained after cranial destruction of experimental juvenile carp and all parasites were maintained in 100% air saturated dechlorinated water at the respective temperatures (i.e. 14 and 24 ± 0.5°C) prior to the infection protocols described below.

#### Protocol 1

Individual juvenile carp (*n* = 128) were experimentally infected while using a dissecting microscope with fibre optic illumination according to King and Cable (2007). In brief, each juvenile carp, under mild anaesthesia (0.02% MS-222), was exposed to two gyrodactylids obtained from sacrificed infected adult carp. This involved bringing a gill filament containing worms near a host and observing parasite transfer onto the operculum or surrounding skin. Fish then recovered from anaesthesia and were returned to tanks. Control fish (*n* = 48) underwent anaesthesia without exposure to infection. All infected and control fish were housed separately, with fish being held either at 14 or 24°C at a density of one fish per 4 L (64 infected + 24 control fish per temperature treatment). A 50% water change occurred weekly throughout the experimental treatment. Experimentally infected fish were screened on days 7, 14 and 38 post-infections (*n* = 10 per time point randomly sampled, per temperature treatment). This involved examining the gills of sacrificed fish and the whole-body surface of hosts using a dissection microscope with fibre optic illumination to determine worm counts, during which sacrificed fish bodies were screened in dechlorinated tap water with a 100% air saturation and gills were screened in separate crystalising glass dishes, also with 100% air saturated water. As no worms were detected in any of the experimentally infected fish screened on days 14 and 38 all remaining fish were sacrificed and screened on day 40 to determine if any infections persisted.

#### Protocol 2

Juvenile carp (*n* = 10) were housed individually with *G. sprostonae* infected gills (i.e. *n* = 45–65 worms per single gill filament) obtained from donor adult fish. All trails were conducted within a small water volume (1 L) maintained at 24°C in dark conditions to facilitate parasite transmission (Brooker et al., 2011). After 24 h fish were euthanised and their whole body and extracted gills examined for parasites via a dissecting microscopy with fibre optic illumination.

#### Protocol 3

To compare the infectivity of *G. sprostonae* between live adult and juvenile carp (at 14 and 24°C), juvenile carp (*n* = 40) were placed in 120 L tanks with an adult showing clinical signs of *G. sprostonae* infection at a density of 5 juveniles per 1 infected adult. After 24 h cohabitation, juveniles were removed from tanks and placed individually in 5 L containers at 14 or 24°C for subsequent infection monitoring. To test the effectiveness of infection through cohabitation, the first batch of juvenile fish (*n* = 5 per temperature treatment) were humanely euthanised, and worm numbers were counted on the body surface and gills immediately after 24 h cohabitation. This confirmed that cohabitation successfully transferred parasites to the body surface and gills of juvenile carp. Three subsequent batches (*n* = 5 per batch and each temperature treatment) were screened non-destructively after 24 h cohabitation and then every alternate day by mildly anesthetising carp (0.02% MS-222) and counting the *G. sprostonae* on the body of juvenile carp. On days 11 and 17, when worm numbers on the body had declined, which was inferred as the point at which parasites had either moved into gills or died, all carp from each temperature treatment were euthanised and parasite numbers on the gills determined.

### 2.3 Statistical analysis

All statistical analyses were performed using RStudio version 1.0.143 (R Studio, 2023). For protocol 1 and 2 due to either only single worms being present (see results) or all worms perishing after experimental infection procedures, no statistical analyses could be performed. Therefore, all the analyses below were conducted on data collected from protocol 3.

A Generalised Linear Model (GLM) with a Gamma error distribution and identity link function was used to analyse the association between persistence of infection (i.e. length of time infections lasted) and the temperature treatment. A Fisher’s exact test was also performed to analyse the difference in the number of fish on which *G. sprostonae* worm numbers increased on the body surface (indicative of parasite reproduction) between fish at 14 and 24°C. To assess whether there was a difference in the total number of worms on the juvenile carp between temperature treatments over the duration of the experiment, we utilised area under curve (AUC) metrics, calculated using the trapezoid rule (White, 2011). A generalised linear mixed model (GLMM) with a negative binomial error distribution and log link function was used to analyse AUC, with temperature treatment and standard length included as a fixed factor and fish ID as a random variable. A GLM with a Poisson error distribution and log link function was utilised to analyse the association between the mean number of parasites counted over the duration of the experiment (17 days) on the total body surface of fish after infections at 14 and 24°C, with host standard length and temperature being fixed factors. To analyse the difference in parasite numbers on the gills of juvenile carp counted on days 1, 11 and 17 post-cohabitations between temperature treatments, a GLM with a Poisson error family and log link function was used, with temperature and host standard length being fixed factors.

All final GLM and GLMM models were chosen based on underlying model assumptions; normality and heteroscedasticity of residuals, lowest AIC values and for Poisson and negative binomial models, the theta parameter, which quantifies overdispersion (Thomas et al., 2015). All final models were refined using stepwise deletion of non-significant variables.

## 3 Results and Discussion

### Protocol 1

At day 7 post-infection, only 2 out of 10 juvenile fish at 24°C were infected with a single gyrodactylid each on the gills, whereas no fish (*n* = 10) were infected at 14°C. All juveniles screened on days 14, 38 and 40 were uninfected at both temperatures. Why juvenile common carp could not sustain gill infections is unclear, but it is possible that common carp are suboptimal hosts. In Iran, *G. sprostonae* hosts included not only common carp but also the silver carp (*Hypophthalmichthys molitrix*) and the big head carp (*H. nobilis*) (see Jalali et al., 2005) and recently this invasive parasite has also been noted in smallmouth yellowfish (*Labeobarbus aeneus*) in southern Africa (Maduenyane et al., 2023). Thus, evidence suggests that *G. sprostonae* is a parasite with low host specificity, which would increase the risk of pathogen spillovers. Furthermore, susceptibility to monogenean infections could be age related as noted in some fish species (e.g. Wunderlich et al., 2022).

### Protocol 2

No *G. sprostonae* worms were found through microscopic analysis of the body surface and gills of juvenile fish (*n* = 10) maintained at 24 h with infected gills filaments from donor adult carp.

### Protocol 3

**Figure 1:**
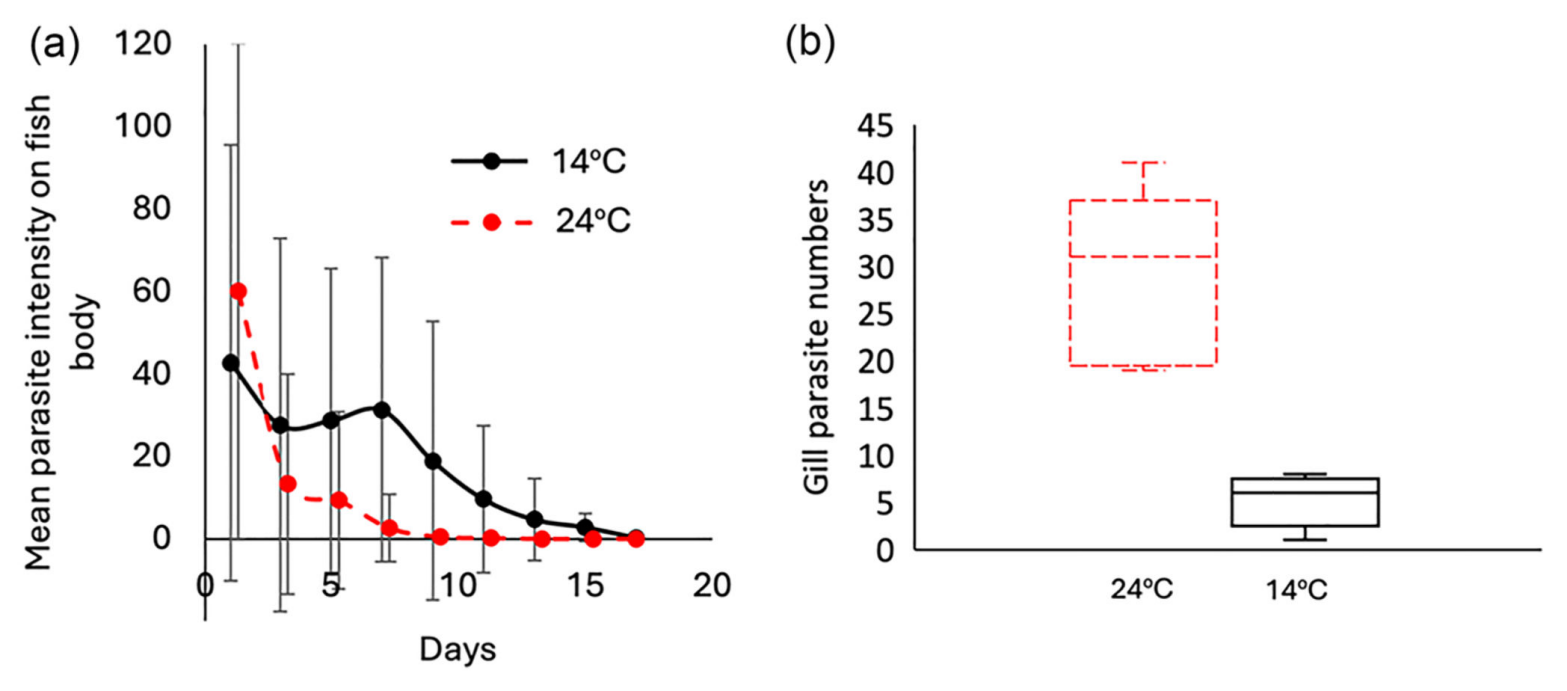
*Gyrodactylus sprostonae* on juvenile carp over 17 days at 24 and 14°C (coloured red and black respectively). (a) Mean parasite intensities on carp body surface (with standard deviation bars) after cohabitation (24 h) with infected adult carp. (b) Box plot showing the median parasite number, inter-quartile range (box) and 1.5x inter-quartile range (whiskers). Parasite numbers on gills of juvenile carp after cohabitation with infected adults followed by sacrificing fish and extracting the gills to count parasites.

**Figure 2:**
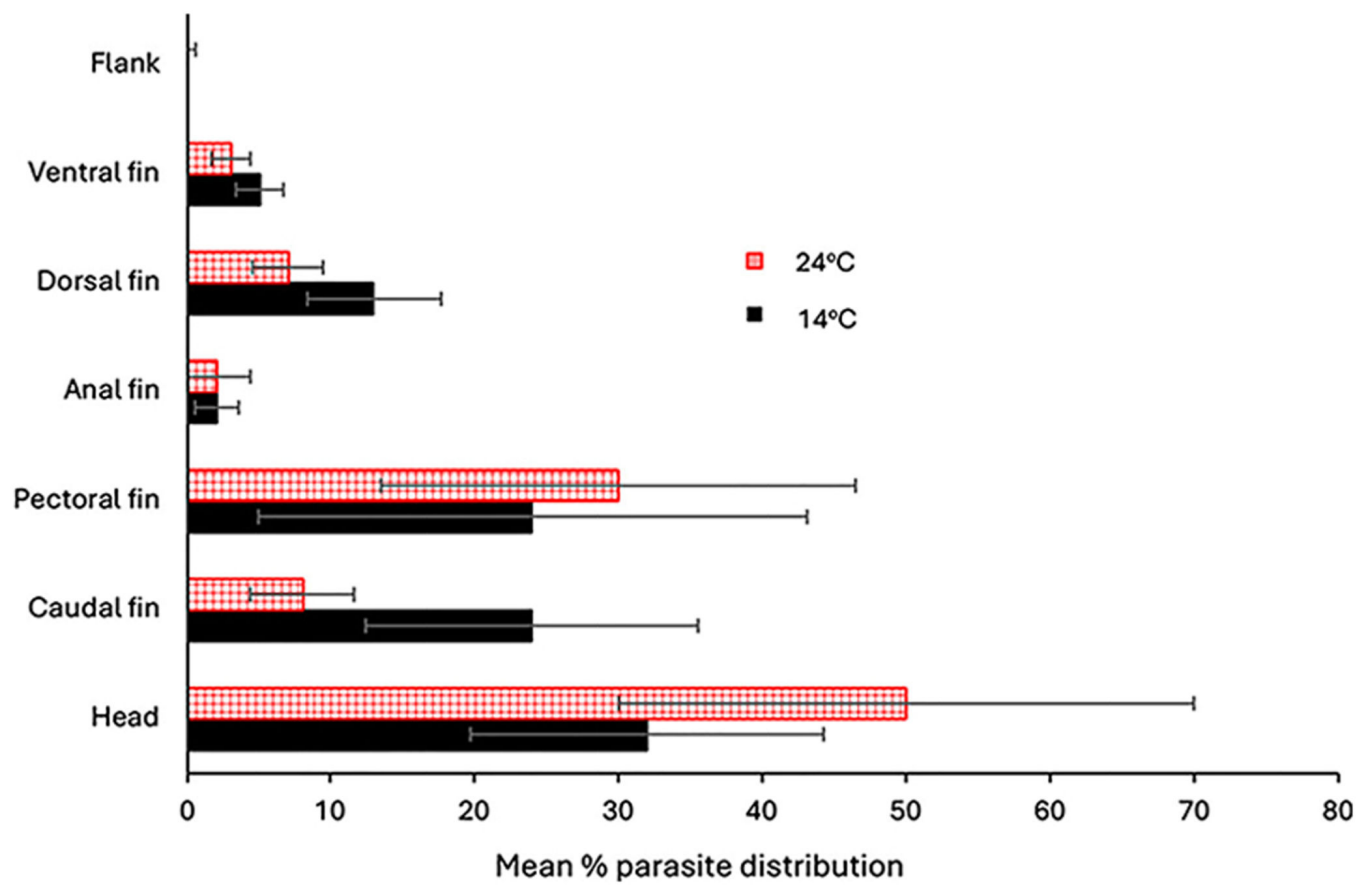
Mean parasite distribution represented as a percentage of total parasite counts on the body surface of the juvenile carp averaged across 17 days of microscopy screening. All data is from Protocol 3 following cohabitation with infected adult carp between temperature treatments (24 and 14°C – coloured red and black respectively) and subsequently screened until all juvenile carp cleared their parasites. Note that the head region includes the gills of fish, where this parasite is mostly aggregated. Also shown are standard deviation bars.

There was a significant difference in total parasite numbers on the body surface of juvenile carp over the duration of the experiment between temperature treatments; fish at 14°C had significantly higher worm numbers than those at 24°C (GLMM of AUC: *Z* = −2.08, SE = .54, *p* = .03, Figure 1a). However, considering total worm numbers on the gills of dissected fish at days 11 and 17, fish at 24°C had significantly higher *G. sprostonae* numbers compared with fish at 14°C (GLM: *Z* = 8.08, SE = .22, *p* < .001, Figure 1b). Infected juvenile standard length (700–900 mm) did not significantly correlate with number of worms at 14 or 24°C (*Z* = 1.12, SE = .03, *p* = .26) and this variable was dropped from the final model. Infections persisted for significantly longer at 14°C on the body surface (maximum of 15–17 days at 14°C compared to 7–9 days at 24°C Figure 1a, GLM: T = 4.05, SE = 1.87, *p* = .001). Considering reproduction on the body surface of carp, at 14°C the number of *G. sprostonae* worms increased marginally on 10 out of 15 fish, whereas at 24°C, parasite numbers only increased on 1 out of 15 carp (Fisher’s exact test, *p* = .001). When considering the parasite numbers on the body and gills, more worms are found in the gills at 24°C, suggesting that at this temperature *G. sprostonae* was more efficient at reaching the gills of carp from the body surface and reproduce. Furthermore, considering the distribution of parasites on the host body, significantly more parasites were aggregated on the head (including gill operculum) and pectoral fins compared to other regions, representing a combined 56% and 80% of the parasite distribution from cold and warm treatments, respectively (GLM: head *Z* = 6.56, SE = .42, *p* < .001; pectoral fin *Z* = 5.62, SE = .42, *p* < .001, Figure 2). Nonetheless, all worms on the gills of all experimentally infected juvenile carp cleared their infections by day 17.

To conclude, regarding *G. sprostonae* infectivity, all juvenile fish, regardless of the infection protocol, failed to sustain infections and all lost their infections by 40 days of monitoring. Second, whilst temperature significantly affected pathogen numbers and infection duration, it did not affect the overall outcome of the trials in terms of parasites being able to establish sustained populations. However, there could be other environmental factors, not tested for this study, that may impact the susceptibility of juvenile carp to this invasive monogenean parasite (see Bakke et al., 2007 for review of environmental factors influencing gyrodactylid infections). Overall, this study supports observations from managed farmed carp populations in that *G. sprostonae* does not appear to be a causal factor of morbidity or mortality in juvenile common carp.

## Data Availability

All data will be made immediately available via Dryad upon publication.
